# Molecular Markers Reveal Limited Population Genetic Structure in a North American Corvid, Clark’s Nutcracker (*Nucifraga columbiana*)

**DOI:** 10.1371/journal.pone.0079621

**Published:** 2013-11-04

**Authors:** Kimberly M. Dohms, Theresa M. Burg

**Affiliations:** Biological Sciences, University of Lethbridge, Lethbridge, Alberta, Canada; University of Innsbruck, Austria

## Abstract

The genetic impact of barriers and Pleistocene glaciations on high latitude resident species has not been widely investigated. The Clark’s nutcracker is an endemic North American corvid closely associated with *Pinus*-dominated forests. The nutcracker’s encompasses known barriers to dispersal for other species, and glaciated and unglaciated areas. Clark’s nutcrackers also irruptively disperse long distances in search of pine seed crops, creating the potential for gene flow among populations. Using the highly variable mitochondrial DNA control region, seven microsatellite loci, and species distribution modeling, we examined the effects of glaciations and dispersal barriers on population genetic patterns and population structure of nutcrackers. We sequenced 900 bp of mitochondrial control region for 169 individuals from 15 populations and analysed seven polymorphic microsatellite loci for 13 populations across the Clark’s nutcracker range. We used species distribution modeling and a range of phylogeographic analyses to examine evolutionary history. Clark’s nutcracker populations are not highly differentiated throughout their range, suggesting high levels of gene flow among populations, though we did find some evidence of isolation by distance and peripheral isolation. Our analyses suggested expansion from a single refugium after the last glacial maximum, but patterns of genetic diversity and paleodistribution modeling of suitable habitat were inconclusive as to the location of this refugium. Potential barriers to dispersal (e.g. mountain ranges) do not appear to restrict gene flow in Clark’s nutcracker, and postglacial expansion likely occurred quickly from a single refugium located south of the ice sheets.

## Introduction

Phylogeography is the study of processes underlying spatial and temporal dimensions of genetic variation [1]. Patterns of genetic variation attained through phylogeographic methods can provide insights into population-level response to disturbance and the processes responsible for historical dispersal and colonization [2,3]. Examining patterns of genetic variation can help us to evaluate the roles of gene flow, bottlenecks, and historical or geological barriers in explaining contemporary patterns of diversity across geographical regions [4]. Contemporary genetic patterns are strongly influenced by postglacial expansion from refugia [5,6], historical and contemporary barriers to dispersal [7,8], and dispersal potential [9].

The repeated glaciations and climate oscillations of the Pleistocene epoch provide a natural tool to address population responses to large-scale landscape changes. Multiple expansions and contractions of ice sheets created a dynamic landscape that repeatedly fragmented historical populations, alternately creating barriers to dispersal and creating new habitats for colonization [10,11]. During the Pleistocene glacial maxima in North America, many populations were confined to refugia (ice-free areas) [10]. Plant and animal species expanded from several known refugia following the retreat of the ice sheets, including Beringia (present-day Bering Sea and parts of Alaska) and three main areas south of the ice sheets (Pacific Coast, Rockies, and Taiga) [10,12]. Additional areas along the periphery of the ice sheets are contested to have been ice-free, such as an Atlantic shelf near present-day Newfoundland [5,10,13].

High latitude resident bird species provide a unique opportunity to investigate patterns of postglacial and barrier-mediated dispersal; historical events shaping current population structure should be particularly evident in these species. Non-migratory species have the potential to retain patterns of genetic variation longer due to limited dispersal, allowing researchers to make inferences about past historic events [14-16]. For example, species of trees show distinct patterns of population genetic structure and have retained information on historic environmental changes [13,15]. As the number of studies on resident species increase, similar patterns are emerging in vertebrate taxa [16-18]. However, other resident species show limited differentiation despite potential barriers to dispersal [19,20].

Clark’s nutcracker (*Nucifraga columbiana*) is a high latitude resident corvid species (Family Corvidae) that inhabits North American coniferous forests. Much of the published work on this species has focused on spatial cognition exhibited by their extensive food caching behaviours [21]. Additional research has concentrated on Clark’s nutcracker’s role as essential seed dispersers for many conifers, particularly *Pinus* species [22-24]. To date, no published phylogeographic work exists for this species, though genetic work has been identified as a priority [21]. Clark’s nutcracker prefers higher altitude montane forests dominated by one or more *Pinus* species. Their close association with pine-dominated forests is due to a specialised diet of pine seeds, which influences dispersal patterns of both nutcrackers and seeds [21,25]. After nestlings fledge, Clark’s nutcrackers have been known to undergo seasonal altitudinal movement to nearby *Pinus-*dominated subalpine areas [21]. In times of food shortage, and occasionally during the non-breeding season, large numbers of birds have been shown to irruptively disperse, leaving home ranges, and travelling more than 100 km in search of large crops of pine seeds [21,26]. Range-wide morphological and plumage variation is well documented for some high latitude resident species [27,28], but there is little evidence of morphological variation in Clark’s nutcracker [21]. Some suggest that this lack of variation is a reflection of high gene flow between populations due to irruptive dispersal, but this has not been investigated to date [21,29].

To address these gaps in phylogeographic knowledge, our goals for this study were to investigate patterns of dispersal and postglacial colonization in Clark’s nutcracker as reflected in genetic structure. We predict that contemporary Clark’s nutcracker populations will exhibit low levels of genetic differentiation between populations due to irruptive food-related dispersal patterns. Mountain ranges and unsuitable habitats will not act as strong barriers to gene flow due to this species’ preference for high altitude sub-alpine habitats [21] where barriers are limited. Rather, dispersal will be restricted by availability of pine-dominated coniferous forests and seed crops. We expect that postglacial colonization likely occurred from a single refugium south of the ice sheets, similar to patterns of postglacial colonization in several North America *Pinus* species [13].

## Materials and Methods

### Ethics statement

All animals captured in the field were handled following animal welfare protocols (#0614 and #1028) approved by the University of Lethbridge Animal Welfare Committee using guidelines set by the Canadian Council on Animal Care (CCAC). Banding in Canada was performed under Canadian Wildlife Service banding permit #10425W (2007) and #10804 (2008 onward) and in the US under US Fish and Wildlife banding permit #23522. All netting and blood sampling was conducted under the appropriate state, provincial, federal, and institutional authorities; detailed permit information is available upon request.

### Sample collection and preparation

**Figure 1:**
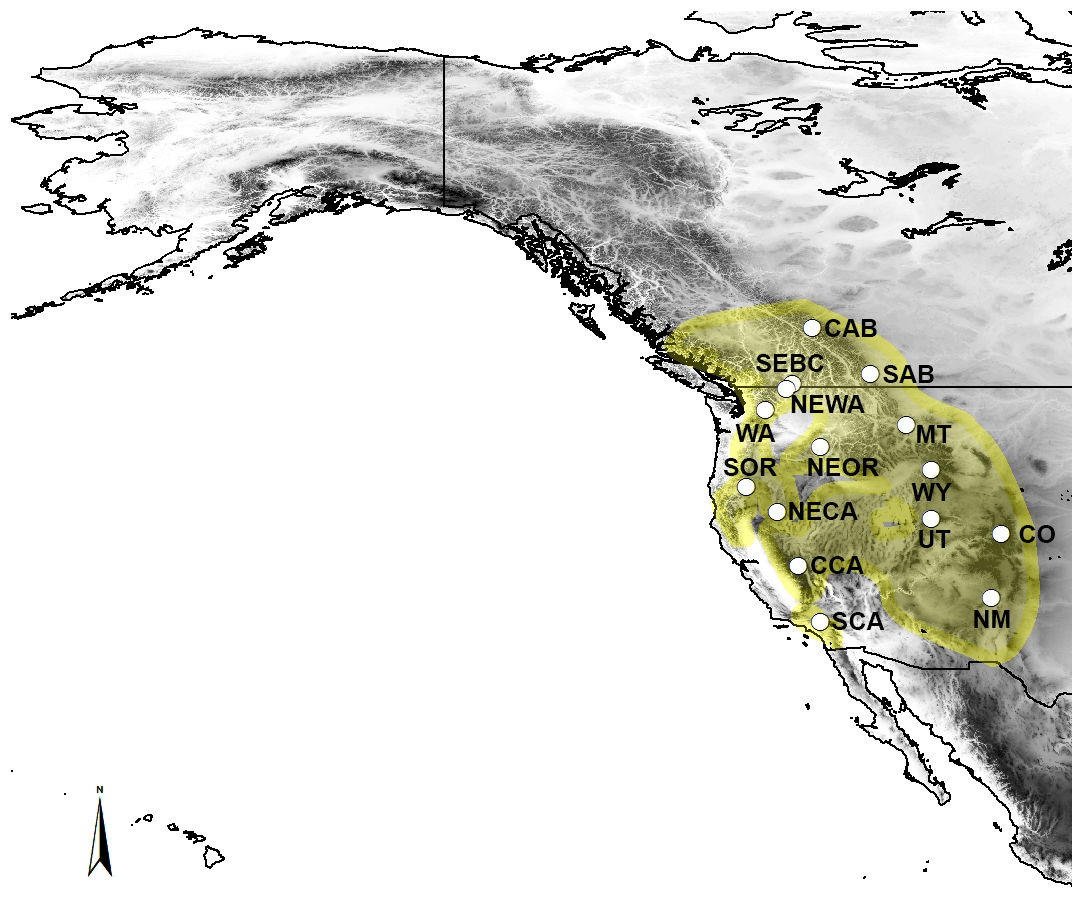
Clark’s nutcracker population sampling locations. Current range overlaid in yellow [76]. Darker shades imply higher elevation areas. See Table 1 for coordinates, sample sizes, and abbreviations per population.

From 2009-2012, we captured up to 30 individual Clark’s nutcrackers at each sampling site (Table 1) using standard mistnetting techniques with call playback. For call playback, we used a medley of group interaction vocalizations sourced from multiple tracks found on xeno-canto.org. Sample collection locations for each defined population (i.e. sampling site) were limited to within a 50 km of radius and were not separated by any obvious barriers to dispersal. We chose sites on either side of possible barriers to dispersal (e.g., Rocky Mountains, Great Basin) and from areas that were previously glaciated and unglaciated during the last glacial maximum (e.g., Central Alberta and New Mexico, respectively; Figure 1). We collected less than 100 μL of blood from each bird by pricking the brachial vein with a sterile needle and collecting blood in a capillary tube. Blood was stored in 95% ethanol and archived at -80°C upon return to the University of Lethbridge. Each bird was banded with a US Fish & Wildlife aluminum band, aged and sexed (if possible), and mass, tarsus, and other morphological measurements taken. Additional tissue and feather samples were obtained from museum collections taken from birds during the breeding season within the past 20 years to ensure all samples were from contemporary populations (Table S1).

**Table 1:** Summary table of and mitochondrial DNA information for populations included in this study.

**Population**	**Lat (N)**	**Long (W)**	***n***	**H_*n*_**	**H_d_**	**π**
CAB	52.687	-118.056	8	6	0.893	0.342
SAB	49.774	-114.397	9	5	0.583	0.127
MT	46.592	-112.158	20	18	0.964	0.281
WY	43.756	-110.581	19	13	0.901	0.295
UT	40.703	-110.612	19	10	0.673	0.226
CO	39.813	-106.195	13	10	0.859	0.221
NM	35.815	-106.852	8	7	0.964	0.396
SCA	34.279	-117.551	14	9	0.912	0.312
CCA	37.771	-118.926	11	9	0.964	0.375
NECA	41.151	-120.205	2	2	1.000	0.456
SOR	42.745	-122.144	12	6	0.849	0.354
NEOR	45.243	-117.524	20	15	0.953	0.402
WA	47.554	-120.958	7	6	0.857	0.329
NEWA	48.845	-119.625	6	4	0.800	0.222
SEBC	49.162	-119.270	1	1	-	-
**Overall**			**169**		**0.854**	**0.324**

Population abbreviations are as follows: CAB = central Alberta, SAB = southern Alberta, MT = Montana, WY = Wyoming, UT = Utah, CO = Colorado, NM = New Mexico, SCA = Southern California, CCA = central California, NECA = northeast California, SOR = southern Oregon, NEOR = northeast Oregon, WA = central Washington, NEWA = northeast Washington, and SEBC = southeast British Columbia. Column headers are: Lat (N) = latitude and Long (W) = longitude of central locations; *n* = number of samples used for mitochondrial DNA analyses; H_n_ = number of haplotypes; H_d_ = haplotype diversity; and π = nucleotide diversity multiplied by 100 for ease of interpretation.

### Laboratory procedures

#### DNA extraction

DNA was extracted from blood, feather, and tissue samples using a modified Chelex protocol [30]. Once extracted, DNA was stored at -20°C in low TE buffer. 

#### Mitochondrial DNA

We amplified a section of the mitochondrial DNA control region (CR) using primers L46 SJ (5’-TTT GGC TAT GTA TTT CTT TGC-3’; Birt & Lemmen, unpublished data) and H1030 JCR 18 [31] corresponding to positions 46 (Domain I) and 1030 (Domain III) of the corvid mitochondrial control region, respectively. Where the complete fragment would not amplify, we used internal primers designed in-house, H560 clnuCR (5’-GCA AAG GGA GGA GTA TGC AG-3’) or L530 corvidCR (5’-CGC CTC TGG TTC CTA TTT CA-3’), with L46 SJ or H1030 JCR 18, respectively, to amplify two overlapping fragments. 

Polymerase chain reactions (PCR) were performed on a Master gradient thermocycler (Eppendorf: Hauppauge, NY) in a 25 μL volume containing 1x goTaq Flexi buffer (Promega: Madison, WI), 2.5 mM MgCl_2,_ 200 μM dNTP, 0.4 μM of each primer, and 0.5 units goTaq Flexi taq polymerase (Promega) under the following conditions: one cycle of 94°C for 120 s, 52°C for 45 s, and 72°C for 60 s, 37 cycles of 94°C for 30 s, 52°C for 45 s and 72°C for 60 s and one cycle of 72°C for five min. PCR products were run on a 0.8% agarose gel to confirm DNA amplification. 

DNA sequencing was performed at McGill University and Génome Québec Innovation Centre on a 3730xl DNA Analyzer (Applied Biosystems: Carlsbad, CA) or at the University of Lethbridge on a 3130 DNA Analyzer (Applied Biosystems). For in-house sequencing we used a shrimp alkaline phosphatase-exonuclease clean up followed by sequencing and sodium acetate precipitation [28] prior to gel electrophoresis. 

#### Microsatellite DNA

Three individuals from geographically distant populations (Montana, southern Oregon, and Colorado) were initially screened for 32 microsatellite primer pairs developed for and used in other corvids. Thirteen additional nutcracker-specific microsatellite loci have since been developed by another independent research group [32]. If screened loci were suspected to be polymorphic and/or amplified clearly, two additional individuals from each of the initial test populations plus three individuals from another population potentially separated by dispersal barriers were screened. Eight loci were polymorphic (Table 2). All forward primers were modified by adding an M13 sequence (5’ – CAC GAC GTT GTA AAA CGA C - 3’) to the 5’ end, which allowed the integration of a fluorescently labeled primer (700 nm or 800 nm) directly into the PCR product. DNA was amplified in a 10 μL reaction with 1x buffer, 1 mM MgCl_2,_ 200 μM dNTP (Fisher Scientific: Hampton, NH), 1 μM of each primer (forward and reverse), 0.05 μM of the fluorescent primer (Eurofins MWG Operon: Huntsville, AL) and 0.5 units taq polymerase under the following conditions: one cycle of 94°C for 120 s, T_1_ for 45 s, and 72°C for 60 s, seven cycles of 94°C for 60 s, T_1_ for 30 s and 72°C for 45 s, 31 cycles of 94°C for 30 s, T_2_ for 30 s, and 72°C for 45 s, and one final elongation cycle at 72°C for five minutes (Table 2).

**Table 2:** Microsatellite primer pairs used in this study.

**Primer**	**Sequence (5' to 3')**	**Buffer**	**taq**	**T_1_**	**T_2_**
ApCo19F	CAG ACT GCA GTC TTG CTA TAG C	Flexi	crimson	45	48
ApCo19R	GCC TTG GAT GCT TTT ACG				
ApCo30F	GCC CTG ATG CTG TTG ATG GT	Flexi	Flexi	45	48
ApCo30R	CTG GAG CCT GGT TTA GAG TTA TGC				
ApCo37F	TGC CAA ATG CAA CCA AAT CTT	Flexi	Flexi	50	52
ApCo37R	CAT CAC TTG CAG AGA GGG CA				
ApCo41F	CCT ACT CTG GGC ACT GTT ATT ATC	crimson	crimson	50	52
ApCo41R	CCC ATT ATC AGC ATG TCG TAC A				
ApCo46F	GGG AGC CTA GTA TGT TAA GAT GCT	Flexi	crimson	50	52
ApCo46R	TTC CAG GTG AGG TGA TTC AGC				
ApCo91F	GTA GTC CCA ATG GTT TCT CTG TC	crimson	crimson	45	48
ApCo91R	GAT GAA GTA ATG TGA AAC CTG G				
PnuA106WF	GTA TTT TGG GAT GTC TTA GGG TTG	Flexi	Flexi	50	52
PnuA106WR	CAC ACT CGA GCT ACA ATA AAC CTG				

Primer names, sequence, focal species, source, and reaction conditions for this study (buffer and taq polymerase, and annealing temperatures, T_1_ and T_2_ (°C)). Forward primers (shown with an ‘F’ suffix) were modified to include a short sequence at the 5’ end allowing for incorporation of the florescent tag. All loci except PnuA106W are sourced from Florida Scrub Jay (*Aphelocoma coerulescens*) [77]. PnuA106W is sourced from Yellow-billed Magpie (*Pica nuttalli*) [78].

PCR products were mixed with a stop solution (95% formamide, 20 mM EDTA and bromophenol blue), denatured for 3 min at 94°C, and run on a 6% polyacrylamide gel using a LI-COR 4300 DNA Analyzer (LI-COR Inc.: Lincoln, NE). Alleles were scored via visual inspection, and genotypes were independently confirmed by a second person. Three controls of known allele sizes (pre-screened individuals) plus a size standard were included on each gel to ensure consistent scoring along with a negative control to ensure no contamination was present.

### Analyses of genetic structure

#### Mitochondrial DNA

Chromatograms were edited and sequences aligned manually in MEGA v5.0 [33]. Shared haplotypes were determined using DnaSP v5.10 [34]. We assessed population structure and relationships among haplotypes using a maximum parsimony network constructed using Network v4.611, which is designed to construct the shortest, least complex network [35]. The median-joining (MJ) tree algorithm was used as this allows for multi-state data (e.g., four nucleotide base options at each variable site), with settings as follows: weight = 10 for each site, epsilon = 0, and the MJ squared option turned off. After the tree was constructed, the MP (maximum parsimony) option was used to remove superfluous links and unnecessary median vectors from the network, which can be produced during the initial network calculations [36].

Genetic variation within populations was measured by calculating haplotype (H_d_) and nucleotide (π) diversity using DnaSP v5.10 [34]. We calculated pairwise Φ_ST_ values (an analogue of Wright’s fixation index (*F*_*ST*_) using Arlequin v3.5.1.3 [37] to examine population structure and test for genetic differentiation among populations and haplogroups. Significance values were corrected using a Benjamini-Hochberg correction [38], to control for false discovery rate (FDR), the expected proportion of falsely rejected hypotheses in multiple significance testing situations where multiplicity might occur.

A Mantel’s test was performed to examine the correlation between genetic and geographic distances in GenAlEx v6.0 [39]. We calculated geographic distances using weighted central coordinates for each population (Table 1) and the Geographic Distance Matrix Generator v1.2.3 [40]. Linearised Φ_ST_ values (Φ_ST_ / 1- Φ_ST_) were used for genetic distances and significance was tested using 9,999 permutations. A mismatch distribution was used to help visualize signatures of demographic expansion, and to test the null hypothesis of historic population growth and expansion [41].

Genetic variation allocated within and among populations was examined using an analysis of molecular variance (AMOVA) in Arlequin v3.5.1.3 [37]. We investigated the potential impact of barriers on genetic variation by performing a spatial analysis of molecular variance (SAMOVA; *K* = 2-12; 100 iterations) [42]; however, the process cannot test for range-wide panmixia as *K* cannot be set to *K* = 1. Alternatively, BAPS v6.0 [43], a Bayesian method for analysing population structure, can detect range-wide panmixia. A preliminary population mixture analysis for spatial clustering of individuals in BAPS used no *a priori* population information and *K*_*max*_ = 13. A subsequent BAPS run was then conducted using *a priori* population information.

#### Microsatellite DNA

Allele frequencies, deviation from Hardy-Weinberg equilibrium (observed (Ho) versus expected (He) heterozygosity), and pairwise *F*_*ST*_ values [44] were calculated with 999 permutations using GenAlEx v6.0 [39] and *P* values were corrected for multiple tests using a Benjamini-Hochberg correction for FDR [38]. Allelic richness was calculated in FSTAT v2.9.3 [45]. A Mantel’s test was performed to test for isolation by distance using the same procedure as for mitochondrial DNA.

Bayesian clustering analyses were conducted using the programs STRUCTURE v2.3.3 [46,47] and BAPS v6.0 [48]. STRUCTURE was run with the following settings: *K* = 1-15, a burn-in of 100,000 followed by 500,000 runs, admixture assumed, correlated allele frequencies and including *a priori* population information 10 replicates were performed for each value of *K*. In STRUCTURE, it can be difficult to decide when *K* captures major structure in the data due to similar lnP(X|K) values, thus two additional analyses were conducted using STRUCTURE results: Bayes factor calculations [46] and Δ*K* [49] using STRUCTURE HARVESTER [50]. One limitation with using STRUCTURE HARVESTER is that it cannot detect when *K* = 1 is the true number of clusters. However, Bayes factors and BAPS are able to detect if *K* = 1, so the addition of these methods is useful in species where panmixia is a possibility. A preliminary population mixture analysis for spatial clustering of individuals in BAPS used no *a priori* population information and *K*_*max*_ = 13. A subsequent BAPS run was then conducted using *a priori* population information.

### Species distribution modeling

#### Sample points

In addition to our sample (field and museum) locations, geo-referenced Clark’s nutcracker locations were obtained from the Global Biodiversity Information Facility (GBIF; http://data.gbif.org/, accessed on 22 January 2013). We excluded occurrences outside of North America, without geo-references, or recorded before 1950 from modeling and further inspected accuracy by plotting points using ArcMap v10.1 (ESRI: Redlands, CA). We removed duplicate records prior to model-building. 

#### Bioclimatic data

Current bioclimatic data were extracted from the WORLDCLIM dataset (v1.4, http://www.worldclim.org/) and LGM bioclimatic data from the Model for Interdisciplinary Research on Climate (MIROC) database [51] at 2.5 min resolution (~4 x 4 km tiles). The current bioclimatic dataset ranges over a 50 year period (1950 - 2000), hence exclusion of observations prior to 1950. Nineteen bioclimatic variables are included in the WORLDCLIM current and LGM dataset [52]. ArcMap v10.1 (ESRI) was used to clip climatic variable layers to include only North America as using smaller geographic areas can improve the predictive power of Maxent models [53]. Prior to constructing SDM, ENMTools v1.3 [54] was used to determine which bioclimatic variables were correlated using R > 0.90 as a cutoff. Nine variables were correlated with at least one other variable and all but one from each set of correlated variables was removed.

#### Maximum entropy (Maxent) distribution modeling

Maxent v3.3.3 [55] was used to model suitable habitat distributions for Clark’s nutcracker at present and during the LGM using hinge features only, regularization multiplier = 1, max number of background points = 10,000, replicate run type of 10 cross-validations, 500 maximum iterations, and 0.00001 convergence threshold. Resulting layers were then exported to ArcMap v10.1, overlayed on a digital elevation map, and processed into images.

## Results

### Genetic analyses

#### Mitochondrial DNA

**Figure 2:**
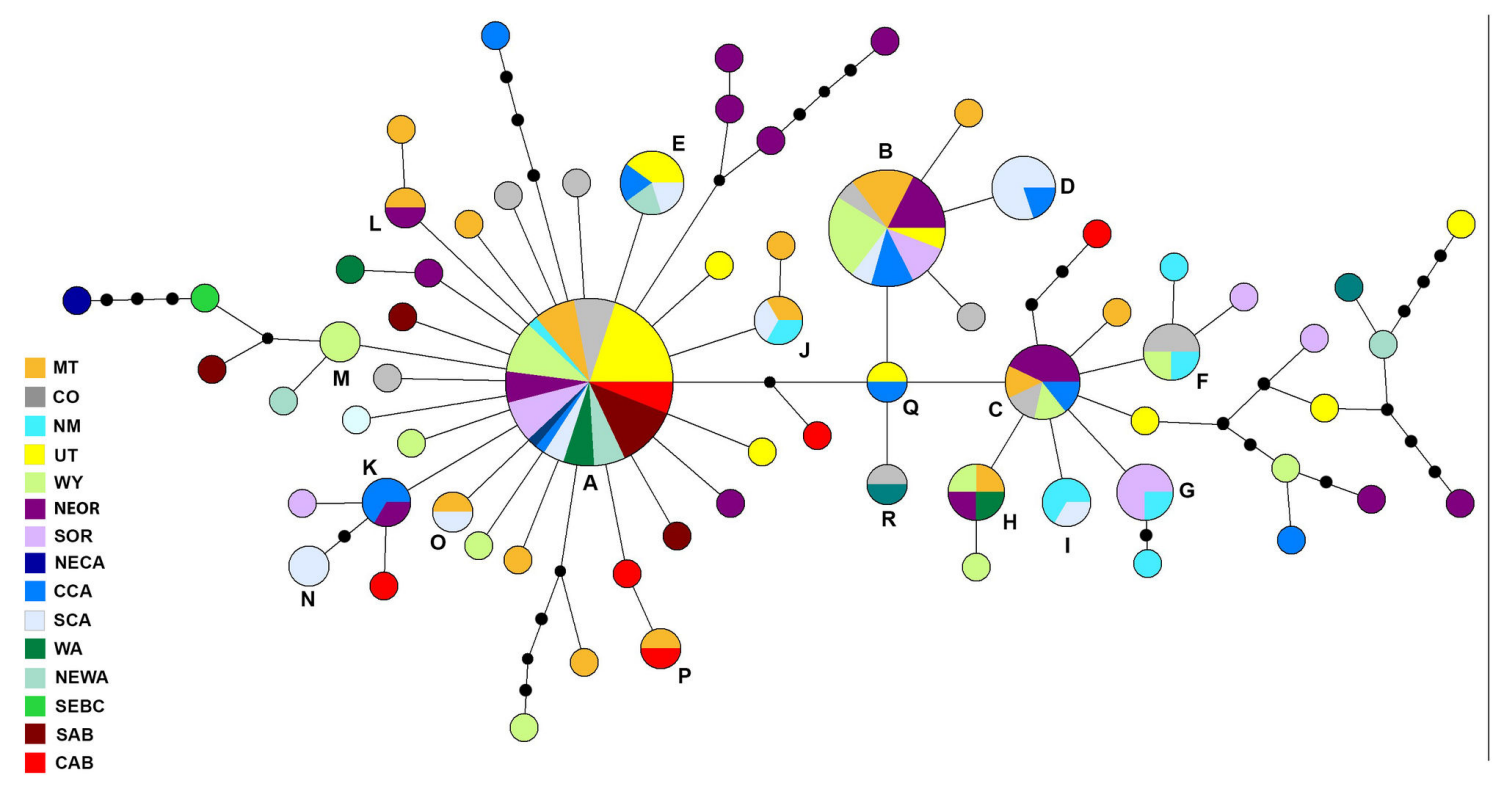
Clark’s nutcracker mitochondrial DNA haplotype network. **Legend**: Maximum parsimony network constructed using a median joining algorithm and post-processed with a maximum parsimony procedure for a 900 base pair fragment of mitochondrial control region from 169 Clark’s nutcrackers across North America. Each colour represents a different population (*n* = 15). Circle size is proportional to number of individuals with that haplotype. Haplotypes represented as pie charts include individuals from multiple populations with pie segments proportional to the number of individuals from each population. Capital letters are those assigned to shared haplotypes (see Table 3). Small black circles represent inferred nodes. See Table 1 for population abbreviations.

Mitochondrial DNA control region sequences (n = 169; Table S1) from 15 populations range-wide revealed 48 variable sites in a 900 base pair (bp) region. In total, 68 haplotypes were recognized in the median-joining analysis (GenBank accession nos. KF687612-KF687679; Figure 2; Table 3). Eighteen haplotypes accounted for 119 individuals; 50 singleton haplotypes were found (Figure 2; Table 3). The largest haplotype group contained a total of 50 individuals from 14 of the 15 populations analysed. Most haplotypes were a single mutational step removed from each other. Overall, limited geographic clustering was observed in the maximum parsimony network (Figure 2). 

**Table 3:** Geographic distribution of shared haplotypes.

**Haplotype**	**CAB**	**SAB**	**MT**	**WY**	**UT**	**CO**	**NM**	**SCA**	**CCA**	**NECA**	**SOR**	**NEOR**	**WA**	**NEWA**	**SEBC**	**Total**
**A**	3	6	4	5	10	4	1	2	1	1	4	3	3	3	-	**50**
**B**	-	-	3	4	1	1	-	1	2	-	2	3	-	-	-	**17**
**C**	-	-	1	1	-	1	-	-	1	-	-	3	-	-	-	**7**
**D**	-	-	-	-	-	-	-	4	1	-	-	-	-	-	-	**5**
**E**	-	-	-	-	2	-	-	1	1	-	-	-	-	1	-	**5**
**F**	-	-	-	1	-	2	1	-	-	-	-	-	-	-	-	**4**
**G**	-	-	-	-	-	-	1	-	-	-	3	-	-	-	-	**4**
**H**	-	-	1	1	-	-	-	-	-	-	-	1	1	-	-	**4**
**I**	-	-	-	-	-	-	2	1	-	-	-	-	-	-	-	**3**
**J**	-	-	1	-	-	-	1	1	-	-	-	-	-	-	-	**3**
**K**	-	-	-	-	-	-	-	-	2	-	-	1	-	-	-	**3**
**L**	-	-	1	-	-	-	-	-	-	-	-	1	-	-	-	**2**
**M**	-	-	-	2	-	-	-	-	-	-	-	-	-	-	-	**2**
**N**	-	-	-	-	-	-	-	2	-	-	-	-	-	-	-	**2**
**O**	-	-	1	-	-	-	-	1	-	-	-	-	-	-	-	**2**
**P**	1	-	1	-	-	-	-	-	-	-	-	-	-	-	-	**2**
**Q**	-	-	-	-	1	-	-	-	1	-	-	-	-	-	-	**2**
**R**	-	-	-	-	-	1	-	-	-	-	-	-	1	-	-	**2**
**Total**	**4**	**6**	**13**	**14**	**14**	**9**	**6**	**13**	**9**	**1**	**9**	**12**	**5**	**4**	**0**	**119**
**Unique**	4	3	7	5	5	4	2	1	2	1	3	8	2	2	1	**50**

Haplotype codes correspond to those found in Figure 2. Population abbreviations and locations are found in Table 1.

**Figure 3:**
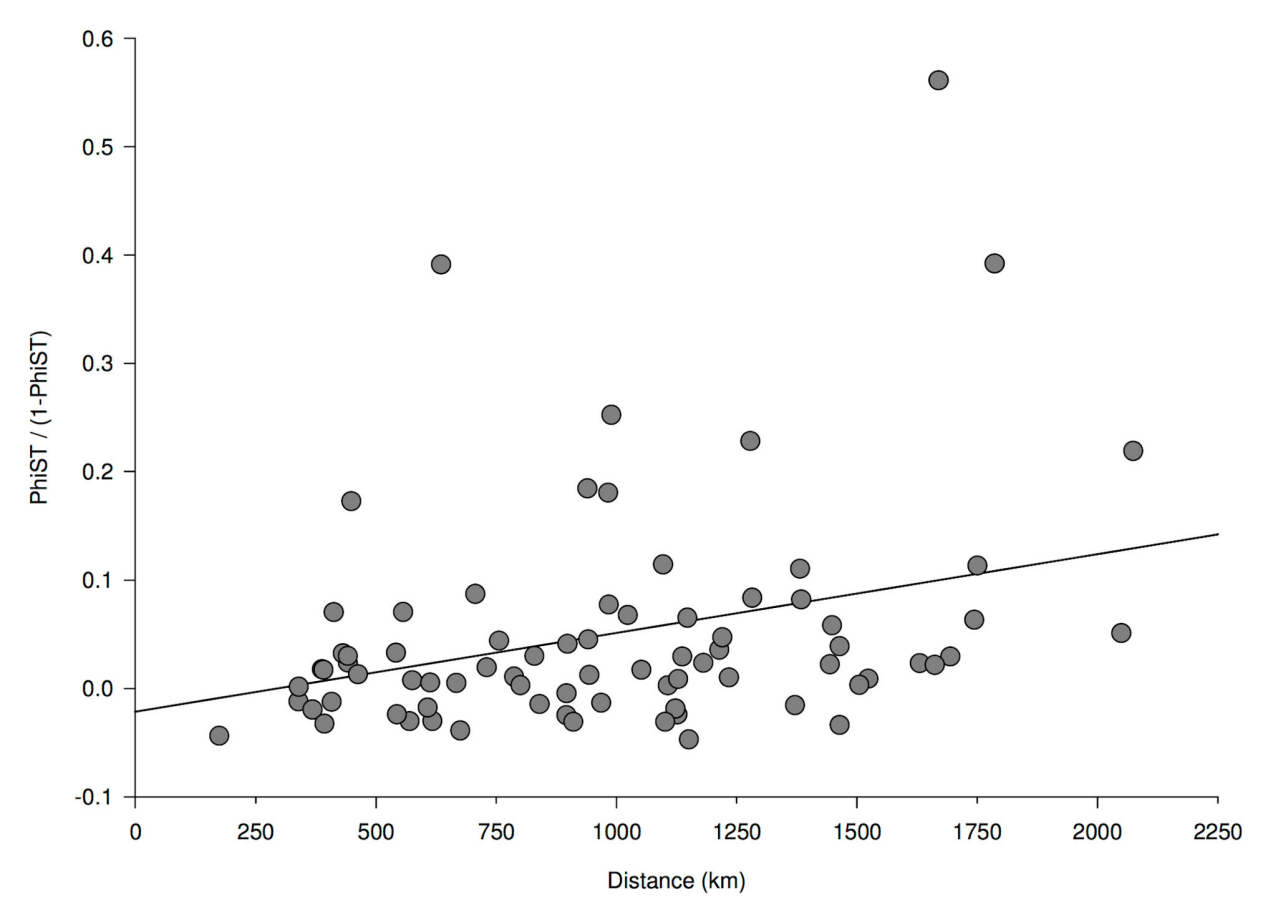
Mantel test of isolation by distance for mitochondrial DNA. **Legend**: *R* ^2^ = 0.082, *P* = 0.040. Each point represents one population pairwise Φ_ST_/ (1- Φ_ST_) plotted against geographic distance between paired populations.

Haplotype diversity (H_d_) values ranged from 0.583 (southern Alberta (SAB)) to 0.964 (Montana (MT), New Mexico (NM), and central California (CCA)) and nucleotide diversity (π) ranged from 0.00127 (SAB) to 0.00402 (northeastern Oregon) for populations with greater than five samples (Table 1). When all samples were combined, overall H_d_ was 0.854, and overall π was 0.00324 indicating high levels of genetic diversity in Clark’s nutcracker. In 78 population pairwise comparisons of genetic differentiation, Φ_ST_ values ranged from -0.050 (*P*_corrected_ = 0.779) for Utah (UT) and northeast Washington (NEWA) to 0.359 (*P*_corrected_ = 0.100) for NM and SAB (Table 4). Most population pairs (57/78) had Φ_ST_ values less than 0.050, though 14 pairs had Φ_ST_ values between 0.050 and 0.150, four pairs had Φ_ST_ values between 0.150 and 0.250, and three pairs had Φ_ST_ values above 0.250. Only four of those Φ_ST_ values were significant after the FDR correction and all occurred between NM and other populations (UT, Wyoming (WY), southern California (SCA), and NEWA). The mismatch distribution did not deviate significantly from the expected signature, so we cannot reject the null hypothesis of historical population growth and expansion [41]. Geographic distance in 78 paired populations ranged from 339 km for WY and UT to 2074 km for NM and central Alberta (CAB). A low, but significant correlation between genetic and geographic distance was detected for mitochondrial DNA (*R* ^2^ = 0.082, *P* = 0.040; Figure 3). When the NM population was removed from the analyses, the relationship between genetic and geographic distance still trended towards isolation by distance, though it was no longer significantly correlated (*R* ^2^ = 0.064, *P* = 0.058; not shown).

**Table 4:** Population pairwise Φ_ST_ values for Clark’s nutcracker mitochondrial DNA.

**MT**	**CO**		**NM**		**UT**		**WY**	**NEOR**	**SOR**		**CCA**		**SCA**		**WA**	**NEWA**		**SAB**	**CAB**	
**MT**				0.832		0.100	0.509	0.714		0.373		0.316		0.509	0.373		0.825	0.530		0.468		0.574
**CO**		-0.026				0.270	0.414	0.883		0.546		0.714		0.771	0.414		0.771	0.373		0.373		0.414
**NM**		0.186		0.147			**<0.001**	**<0.001**		0.234		0.509		0.289	**<0.001**		0.373	**<0.001**		0.100		0.234
**UT**		0.005		0.017		**0.281**		0.589		0.296		0.289		0.509	0.296		0.714	0.779		0.473		0.270
**WY**		-0.012		-0.031		**0.156**	0.001			0.462		0.509		0.771	0.373		0.771	0.654		0.479		0.414
**NEOR**		0.023		0.003		0.099	0.042	0.007				0.383		0.756	0.270		0.756	0.414		0.289		0.414
**SOR**		0.039		-0.016		0.009	0.072	0.012		0.013				0.779	0.373		0.714	0.373		0.100		0.366
**CCA**		0.008		-0.025		0.102	0.011	-0.014		-0.015		-0.031			0.654		0.771	0.513		0.270		0.456
**SCA**		0.022		0.023		**0.202**	0.043	0.034		0.045		0.063		-0.013			0.691	0.499		0.334		0.366
**WA**		-0.041		-0.035		0.102	-0.019	-0.032		-0.020		-0.025		-0.032	0.003			0.771		0.373		0.714
**NEWA**		0.005		0.037		**0.282**	-0.050	-0.005		0.031		0.080		0.010	0.023		-0.046			0.876		0.509
**SAB**		0.017		0.077		0.359	0.017	0.019		0.066		0.153		0.076	0.059		0.032	-0.034				0.289
**CAB**		0.003		0.028		0.180	0.055	0.028		0.029		0.061		0.021	0.048		-0.018	0.029		0.066		

Φ_ST_ values are below diagonal, significance values corrected for false discovery rate above diagonal. Values are based on 110 permutations for mitochondrial DNA control region in 166 Clark’s nutcrackers from 13 populations (n ≥ 5) in North America. Bolded values indicate significance. See Table 1 and Figure 1 for sampling locations and abbreviations.

Both AMOVA and SAMOVA showed that most genetic variation is contained within rather than among population groups. The highest value of between-group variation was observed when *K* = 1 for AMOVA analyses (3.4% between groups, 96.6% within groups) and for *K* = 2 in SAMOVA (17.8% between groups, 80.7% within groups), with one group consisting of NM and the other of all other populations pooled. The preliminary (no *a priori* population information) and secondary (using *a priori* population information) BAPS analyses for spatial clustering of individuals found no significant differentiation between individuals or populations (i.e., *K* = 1). 

#### Microsatellite DNA

A total of seven polymorphic loci (Table 5) were used for analyses; an eighth locus (ApCo37) was removed due to limited amplification success and subsequent missing data (~50%). We used the thirteen populations with greater than five samples for microsatellite analyses (Table 1; Table S1). The total number of alleles detected ranged from four in PnuA106W to 10 in ApCo40 with an average of 6.43. Only 13 of the 91 loci-population comparisons showed significant deviations from Hardy-Weinberg and all showed lower heterozygosity than expected. ApCo41 has a significant heterozygote deficit in five of the 13 populations sampled and while two of these (NEWA and CAB) had fewer samples (*n* ≤ 8), the others had a larger number of samples (*n* ≥ 14; Table 5). 

**Table 5:** Summary table of seven microsatellite loci used to analyze 13 Clark’s nutcracker populations.

	**ApCo19**	**ApCo30**	**ApCo40**	**ApCo41**	**ApCo46**	**ApCo91**	**PnuA106W**
**Central Alberta** (n=8)							
**A_n_**	3	3	5	2	4	4	2
**A_r_**	2.359	2.361	3.160	1.692	2.967	2.963	1.429
**Ho**	0.571	0.625	0.750	0.000	0.429	0.625	0.143
**He**	0.500	0.555	0.625	0.245	0.602	0.648	0.133
**P**	ns	ns	ns	**	ns	ns	ns
**Southern Alberta** (n=12)							
**A_n_**	3	3	5	2	6	2	2
**A_r_**	2.190	2.274	3.039	2.000	4.018	1.846	1.908
**Ho**	0.500	0.400	0.583	0.667	0.750	0.143	0.417
**He**	0.497	0.535	0.611	0.444	0.806	0.337	0.413
**P**	ns	ns	ns	Ns	ns	ns	ns
**Montana** (n=25)							
**A_n_**	3	3	6	4	5	5	3
**A_r_**	2.142	2.566	3.430	1.835	3.446	3.062	1.878
**Ho**	0.640	0.440	0.840	0.320	0.783	0.750	0.320
**He**	0.486	0.586	0.734	0.282	0.738	0.684	0.351
**P**	ns	*	ns	Ns	ns	ns	ns
**Wyoming** (n=30)							
**A_n_**	3	4	9	3	5	3	3
**A_r_**	2.022	2.510	3.937	1.620	2.686	2.314	1.800
**Ho**	0.483	0.357	0.724	0.136	0.550	0.400	0.241
**He**	0.463	0.543	0.793	0.208	0.556	0.460	0.313
**P**	ns	ns	**	***	*	*	ns
**Utah** (n=20)							
**A_n_**	2	4	6	3	5	3	3
**A_r_**	1.962	2.558	3.247	1.891	2.926	2.426	1.862
**Ho**	0.579	0.294	0.650	0.316	0.471	0.571	0.278
**He**	0.478	0.528	0.700	0.342	0.619	0.538	0.323
**P**	ns	***	ns	Ns	*	ns	ns
**Colorado** (n=13)							
**A_n_**	3	3	6	2	5	4	3
**A_r_**	2.247	2.353	3.509	1.273	3.643	2.632	2.062
**Ho**	0.385	0.417	0.769	0.091	0.750	0.444	0.385
**He**	0.462	0.517	0.728	0.087	0.760	0.512	0.411
**P**	ns	ns	ns	Ns	ns	ns	ns
**New Mexico** (n=9)							
**A_n_**	3	3	5	2	4	3	2
**A_r_**	2.241	2.317	3.452	1.867	3.084	2.467	1.975
**Ho**	0.667	0.778	0.750	0.400	0.429	0.400	0.111
**He**	0.475	0.549	0.719	0.320	0.663	0.460	0.475
**P**	ns	ns	ns	Ns	ns	ns	*
**Southern California** (n=14)							
**A_n_**	3	3	7	3	5	4	2
**A_r_**	2.111	2.375	3.615	1.646	3.265	2.645	1.976
**Ho**	0.429	0.571	1.000	0.077	0.571	0.636	0.286
**He**	0.457	0.487	0.737	0.210	0.694	0.566	0.490
**P**	ns	ns	ns	**	ns	ns	ns
**Central California** (n=11)							
**A_n_**	3	3	7	2	4	4	3
**A_r_**	1.909	2.231	4.233	1.834	3.173	2.961	2.247
**Ho**	0.364	0.545	0.636	0.273	0.800	0.636	0.636
**He**	0.310	0.517	0.818	0.351	0.685	0.665	0.533
**P**	ns	ns	ns	Ns	ns	ns	ns
**Southern Oregon** (n=12)							
**A_n_**	2	5	6	3	4	4	2
**A_r_**	1.940	2.894	3.759	2.202	2.843	2.645	1.908
**Ho**	0.500	0.750	1.000	0.300	0.750	0.417	0.417
**He**	0.444	0.597	0.760	0.405	0.618	0.549	0.413
**P**	ns	ns	ns	Ns	ns	ns	ns
**Northeast Oregon** (n=20)							
**A_n_**	2	3	8	3	6	4	2
**A_r_**	1.970	2.681	3.294	1.891	3.798	2.406	1.764
**Ho**	0.450	0.600	0.700	0.105	0.750	0.500	0.100
**He**	0.489	0.625	0.704	0.342	0.790	0.510	0.320
**P**	ns	ns	ns	**	ns	ns	**
**Washington** (n=7)							
**A_n_**	2	3	7	2	3	3	3
**A_r_**	1.992	2.622	4.045	1.500	2.758	2.682	2.359
**Ho**	0.500	0.571	0.857	0.167	0.500	0.500	0.714
**He**	0.486	0.571	0.765	0.153	0.625	0.569	0.500
**P**	ns	ns	ns	Ns	ns	ns	ns
**Northeast Washington** (n=6)							
**A_n_**	2	3	4	2	5	3	2
**A_r_**	1.998	2.545	2.969	1.773	3.924	2.545	1.992
**Ho**	0.333	0.667	0.667	0.000	0.833	0.500	0.833
**He**	0.500	0.500	0.625	0.278	0.764	0.500	0.486
**P**	ns	ns	ns	*	ns	ns	ns
**Overall**	A_n_ = 10	A_n_ = 5	A_n_ = 10	A_n_ = 7	A_n_ = 7	A_n_ = 7	A_n_ = 4

Only populations with greater than five samples were used; *n* = number of samples used in genotyping and analyses; A_n_ = number of alleles; A_r_ = allelic richness; Ho = observed and He = expected heterozygosity; P = departures from Hardy-Weinberg equilibrium (ns = not significant, *P < 0.05, **P < 0.01, ***P < 0.001. See Table 1 for population locations.

**Figure 4:**
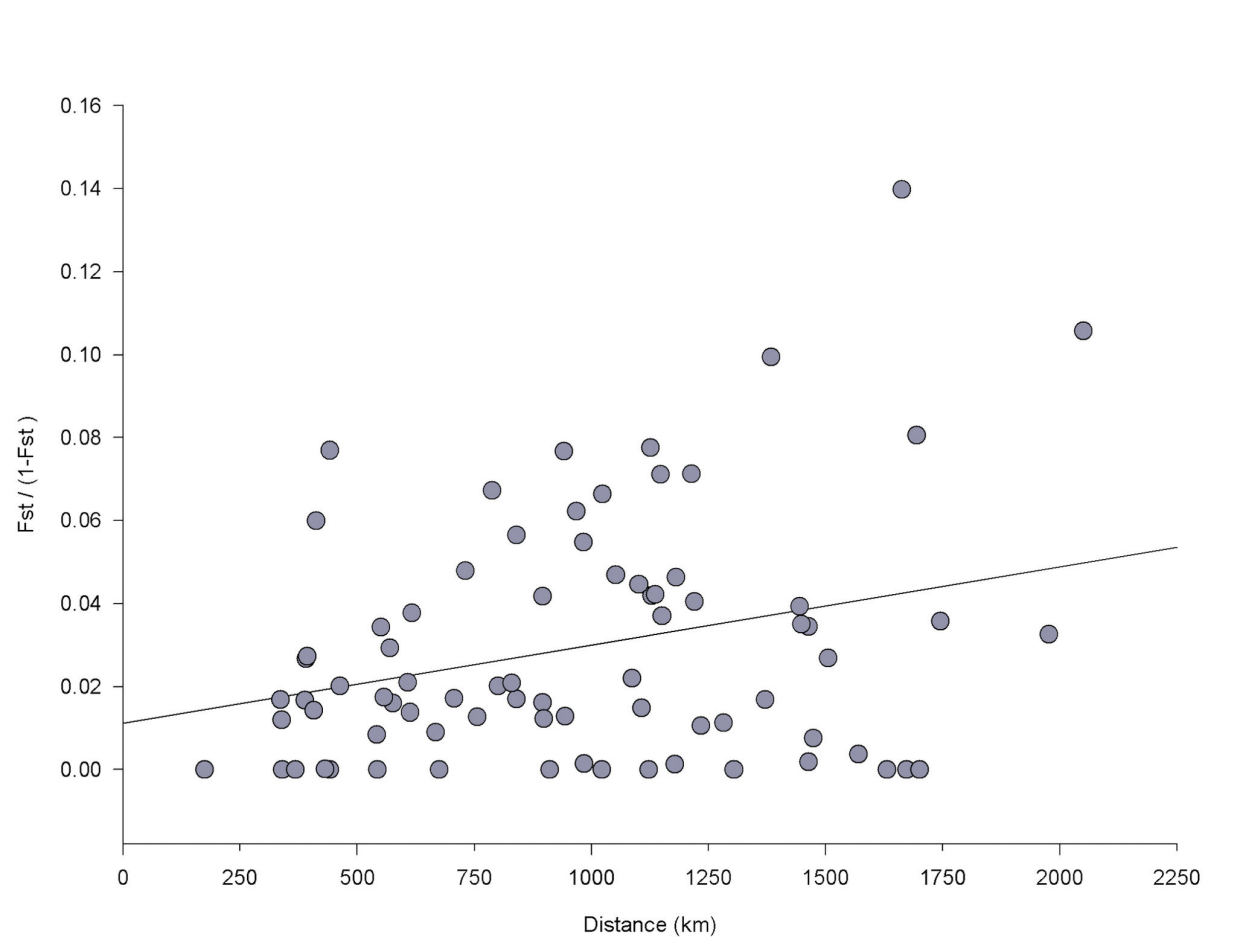
Mantel test of isolation by distance for seven microsatellite loci. **Legend**: *R* ^2^ = 0.078, *P* = 0.029. Each point represents one population pairwise *F*_*ST*_ / (1- F_*ST*_) plotted against straight line geographic distance between paired populations.

Limited population differentiation was detected with a global *F*_*ST*_ value of 0.070. Paired *F*_*ST*_ values ranged from 0.000 (14/78 comparisons) to 0.123 (CCA and central Alberta (CAB); Table 6). After Benjamini-Hochberg FDR correction, 21 of 78 paired comparisons were significant (Table 6). A weak, but significant, correlation was found between genetic and geographic distance using Mantel’s test (*R* ^2^ = 0.078, *P* = 0.029; Figure 4).

**Table 6:** Population pairwise *F*_*ST*_ values for Clark’s nutcracker.

	**MT**	**CO**	**NM**	**UT**	**WY**	**NEOR**	**SOR**	**CCA**	**SCA**	**WA**	**NEWA**	**SAB**	**CAB**
**MT**		0.185	0.454	0.205	0.100	0.454	0.210	**0.032**	**0.028**	0.454	0.311	0.093	0.196
**CO**	0.016		0.238	0.189	**0.035**	0.189	0.205	0.017	**0.035**	0.454	0.189	0.268	**0.016**
**NM**	0.001	0.017		0.084	0.189	0.454	0.378	0.196	0.454	0.454	0.454	0.414	0.189
**UT**	0.009	0.016	0.033		0.454	0.189	0.454	**0.023**	**0.016**	0.454	0.142	**0.017**	0.093
**WY**	0.012	**0.028**	0.017	0.000		0.081	0.189	**0.016**	**0.016**	0.454	0.072	**0.017**	**0.035**
**NEOR**	0.000	0.015	0.000	0.012	0.016		0.167	**0.024**	**0.050**	0.454	0.454	0.189	0.210
**SOR**	0.012	0.017	0.007	0.001	0.013	0.020		0.058	**0.024**	0.467	0.293	**0.031**	**0.032**
**CCA**	**0.040**	**0.072**	0.021	**0.063**	0.059	**0.054**	0.036		0.234	0.093	0.394	**0.017**	**0.016**
**SCA**	**0.038**	**0.044**	0.000	**0.071**	**0.066**	**0.039**	**0.062**	0.014		0.196	0.454	0.093	**0.024**
**WA**	0.000	0.002	0.000	0.000	0.000	0.000	0.000	0.043	0.026		0.454	0.358	0.267
**NEWA**	0.014	0.033	0.000	0.036	0.040	0.000	0.017	0.010	0.000	0.000		0.196	0.072
**SAB**	0.026	0.011	0.004	**0.045**	**0.046**	0.017	**0.052**	**0.090**	0.034	0.008	0.027		**0.037**
**CAB**	0.020	**0.075**	0.032	0.034	**0.041**	0.020	**0.066**	**0.123**	**0.096**	0.021	0.071	**0.057**	

*F*_*ST*_ values are below diagonal and significance values corrected for false discovery rate are above diagonal. Values are based on 110 permutations using seven polymorphic microsatellite loci in 187 Clark’s nutcrackers from 13 populations in North America. See Table 2 and Figure 1 for population abbreviations.

**Figure 5:**
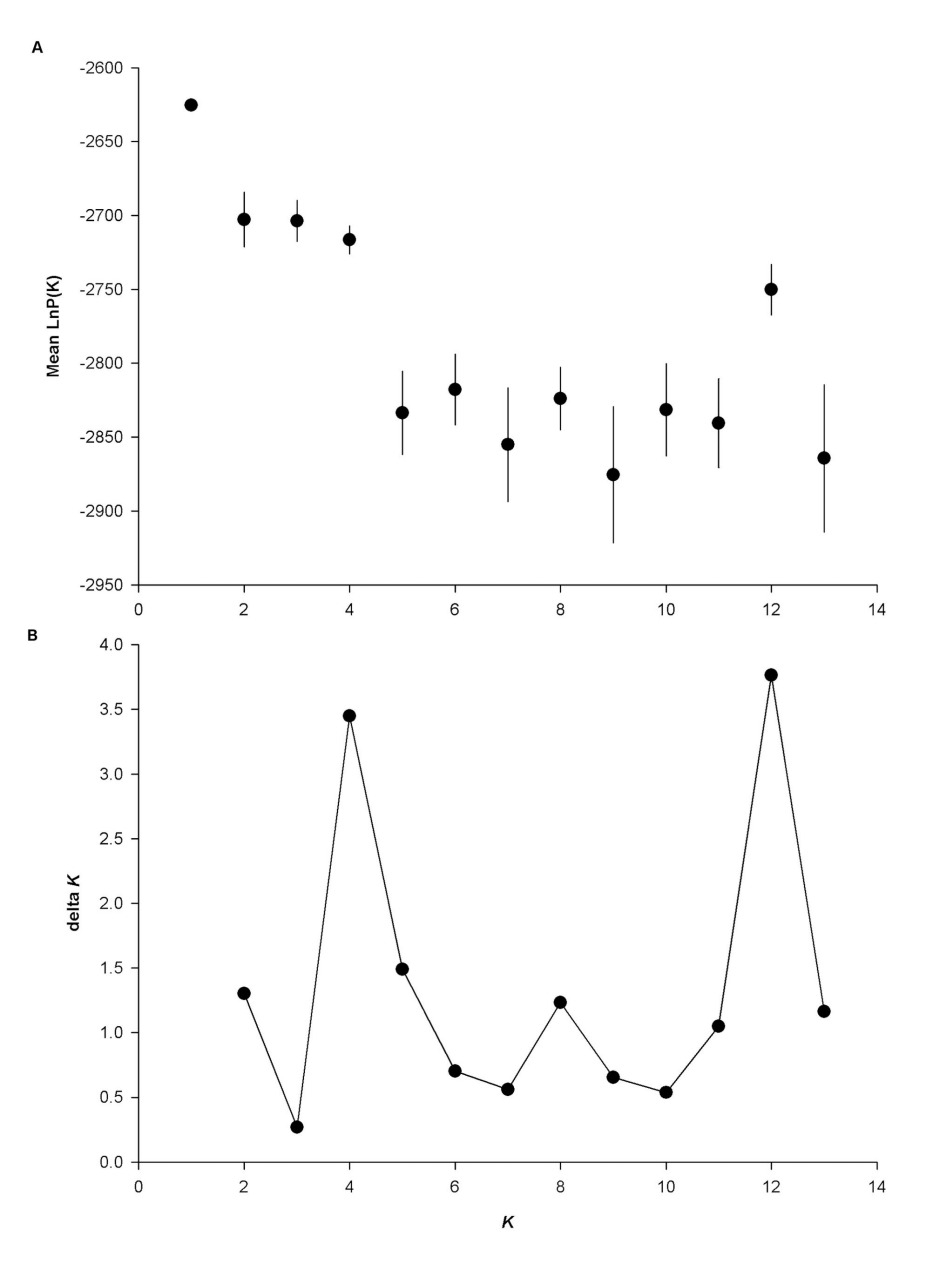
Measures of optimal *K* values from STRUCTURE analyses. **Legend**: A) Penalised log likelihood test (mean ± SE) and B) ∆K depicting clusters (K). The penalised log likelihood test takes the maximum ln Pr (X | K) as the correct number of clusters and ∆K infers the number of clusters from the difference between ln Pr (X | K).

STRUCTURE results suggested that the optimal number of groups was *K* = 1 (Figure 5A). Further analyses produced peaks at *K* = 1, *K* = 4, and *K* = 12 (Figure 5B), though *K* = 1 produced the highest peak and highest posterior probabilities (mean (Pr (K=1)) = -2625.45) and is supported by Bayes factor values (Pr (K=1) = 1) and BAPS results. 

#### Species distribution modeling

A total of 1563 presence records were used for training and 174 for testing the distribution models. After removing correlated bioclimatic layers, 10 layers were used for model construction (bio1-4, 8, 12, 14-15, 18-19). Mean area under the receiver operating curve (AUC) was 0.907 (range = 0.906 - 0.908), suggesting that the current model was highly suitable for backcasting to the paleodistribution model. Isothermality (mean diurnal temperature range/mean annual temperature range (bio3)), temperature seasonality (bio4), and mean annual temperature (bio1) were the most important variables contributing to the model at 44.3%, 37.7%, and 11.1% respectively. 

**Figure 6:**
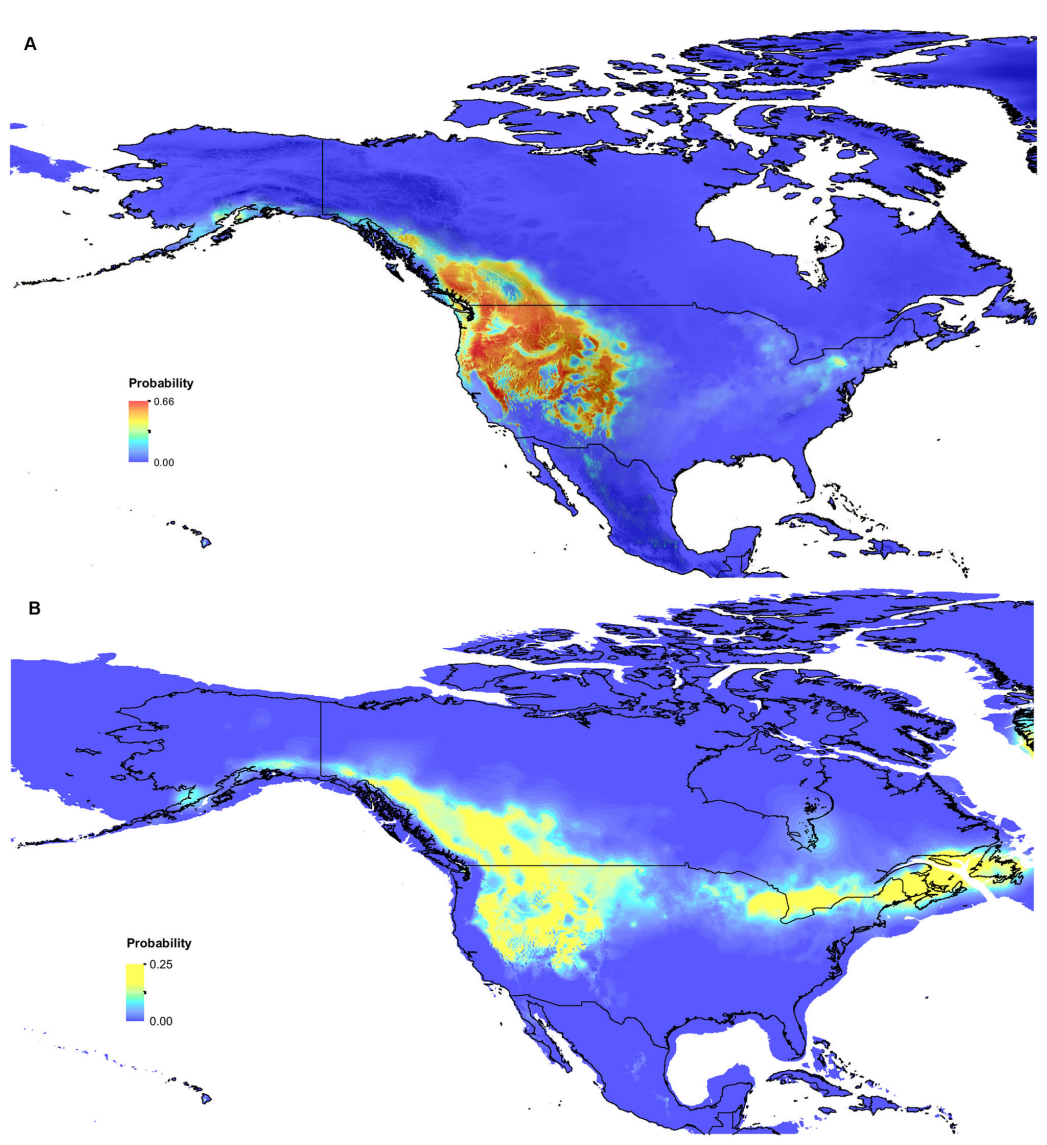
Current and paleodistribution models of suitable Clark’s nutcracker habitat in North America. **Legend**: Probability of suitable Clark’s nutcracker habitat at A) present (overlaid on digital elevation model and B) during last glacial maximum (~21 kya), overlaid on current North American boundaries generated by species distribution modeling. In B), blue areas outside of current coastlines represent historical extent of terrestrial landmass.

The current distribution predicted by the Maxent model (Figure 6A) closely approximated the known Clark’s nutcracker range in North America (Figure 1). The model returned a maximum 66% probability of suitable habitat throughout the range. The paleodistribution model returned a maximum 25% probability of predicted suitable habitat, mostly concentrated in the western mountainous regions and along the southeast edge of the ice sheets during the last glacial maximum (Figure 6B).

## Discussion

### Population genetic structure

Using a combination of measures of population differentiation and clustering analyses, our study suggests high levels of population connectivity between most Clark’s nutcracker populations in North America. We found limited differentiation in mitochondrial DNA (Table 4), with only the New Mexico population showing significant pairwise differences from four other populations. Microsatellite data indicate higher levels of population differentiation between Clark’s nutcracker populations, though this predominantly occurs between populations at the edges of the species’ range (e.g. CAB and SCA; Table 6). Geographic distance rather than barriers appear to influence mitochondrial and nuclear gene flow, as there is a weak, but significant effect of isolation by distance on nutcracker populations for both molecular markers (Figures 4 and 5). However, for mitochondrial DNA, this pattern seems to be driven by populations in New Mexico, suggesting limited peripheral isolation for this marker. For microsatellite data, additional peripheral populations (CAB, SAB, and SCA) are significantly differentiated in multiple comparisons (Table 6), suggesting peripheral isolation in addition to isolation by distance. Clustering analyses do not support strong population differentiation for either nuclear or mitochondrial markers, with a single population being the best fit for the data in both cases. In the case of microsatellite data, these results should be interpreted with caution as both STRUCTURE and BAPS may struggle with situations where differentiation is low (*F*_*ST*_ < 0.030) [56], though global values (*F*_*ST*_ = 0.070) suggest this is likely not the case. 

High levels of haplotype, nucleotide, and allelic diversity throughout most nutcracker populations suggest large population sizes and few founder effects or population bottlenecks. The exception to this is the southern Alberta population, which exhibits reduced haplotype and nucleotide diversity for mitochondrial markers relative to the rest of the populations, though microsatellite loci have high levels of allelic richness and heterozygosity (Table 5). This difference between marker genetic diversity may be due to a historical founder effect in previously glaciated southern Alberta (mtDNA), with nuclear gene flow being less limited (microsatellites). 

Patterns of potential panmixia and limited population differentiation are not unique to Clark’s nutcracker. Many other species that undergo irruptive and seasonal dispersal also exhibit limited population differentiation despite the presence of proposed barriers to dispersal. Studies of *Pinus* seed specialists, such as pygmy nuthatch (*Sitta pygmaea*) [57], crossbill species (*Loxia* *spp.*) [19], and Clark’s nutcracker’s sister species, Eurasian nutcracker (*Nucifraga caryocatactes*) [58], found no significant genetic differentiation among populations despite potential barriers to dispersal. Limited differentiation has been found in other species with irruptive or seasonal dispersal, such as boreal owl (*Aegolius funereus*) [59], and European redpoll species (*Carduelis* *spp.*) [60], yet Steller’s jay (*Cyanocitta stelleri*) [14] and white-breasted nuthatch (*Sitta carolinensis*) [61] exhibit significant differentiation between populations and limited gene flow corresponding with barriers to dispersal. In other taxa, peripheral populations are more likely to be isolated due to reduced gene flow [16,62,63], while maintaining panmixia in the majority of the range [64]. Overall, Clark’s nutcracker population connectivity seems to be primarily limited by distance with some peripheral isolation at the southern and northern edges of the range, which is reasonable for a high elevation species with irruptive and seasonal altitudinal dispersal.

### Postglacial colonization

After the LGM, species that rapidly expanded from a single refugium with high levels of gene flow are expected to exhibit three characteristics: 1) a star-like phylogeny with many low frequency single haplotypes separated from high frequency central ancestral haplotypes by few mutational steps [65]; 2) low levels of genetic subdivision between and within populations [4,66]; and 3) a mismatch distribution of pairwise differences among haplotypes indicating a sudden increase in expansion from a single population [67]. These signals of expansion from a single refugium have been reported in several North American bird species (e.g., multiple species reviewed in 68, downy woodpecker (*Picoides pubescens*) [20]) as well as many plant species, including several *Pinus* species that Clark’s nutcracker specializes upon (reviewed in 13). Clark’s nutcracker mitochondrial DNA exhibits a star-like phylogeny with one major shared haplotype, and short connections between this haplotype and others (Figure 2), in addition to a modeled paleodistribution that suggests that this species expanded from a single refugium after the LGM. This pattern is less complex than that found in whitebark pine postglacial expansion: colonization is thought to have occurred from three refugia (Oregon, Idaho, and Colorado) [25]. Given that Clark’s nutcrackers range extends farther south than that of whitebark pine, nutcrackers may have been isolated in a more southern refugium and expanded into whitebark pine refugia from there, subsequently assisting with seed distribution northward. 

In addition to the aforementioned characteristics, temperate species that expanded north from a southern refugium have been shown to exhibit genetic differentiation between northern and southern populations corresponding to previously unglaciated and glaciated areas [12]. Southern populations were less impacted by glaciation and should show higher levels of nucleotide diversity and phylogeographic structure compared to northern populations [69]. This pattern occurs to varying degrees in *Pinus* species on which Clark’s nutcracker commonly feed: whitebark pine (*Pinus albicaulis*) [25], white pine (*P. monticola*) [70], limber pine (*P. flexilis*) [71,72], and Ponderosa pine (*P. ponderosa*) [73,74]. However, this pattern does not distinctly appear in Clark’s nutcracker. All southern and northern populations sampled exhibit high levels of heterozygosity, nucleotide and allelic diversity, and limited differentiation from other populations, making it very challenging to pinpoint a single refugium for this species. In addition, while species distribution models for current suitable nutcracker habitat performed very well (high AUC values), paleodistribution models returned reduced probabilities (< 30%) of suitable LGM habitat throughout unglaciated areas. Taken together, these results point to a single refugium or multiple small, connected refugia, the location of which is not obvious through genetic signatures or paleodistribution modeling.

### Conclusions and future research

Throughout its range, Clark’s nutcracker exhibits low levels of population differentiation and does not appear to be limited by potential barriers to dispersal, though peripheral populations may be slightly isolated. Future research could integrate additional mitochondrial markers to elucidate the location of a glacial refugium or refugia for this species. Mitochondrial markers with lower rates of mutation in corvids (e.g. cytochrome B) [31] may retain historic genetic signals longer and thus provide additional historic information to this study. Samples at the northern and isolated southern extremes of the range may also increase our understanding of peripheral isolation and postglacial colonization for this species. Given the importance of Clark’s nutcracker to dispersal of *Pinus* species in North America [21,22] and current conservation and management concerns for many *Pinus* species [75], further understanding of nutcracker dispersal may also assist in future forest management and recovery plans.

## Supporting Information

Table S1 **Summary table of Clark’s Nutcracker samples used in analyses.** Sequence (mtDNA): Y = successfully sequenced. N = not sequenced. Genotyped (SSR): Y = successfully genotyped. N = not genotyped. Sources include Burg lab (wild), TS = T. Schaming, The Field Museum (TFM), Burke Museum at the University of Washington (UWBM), Museum of Southwest Biology (MSB), Louisiana State University Museum of Natural Sciences (LSU), American Museum of Natural History (AMNH), Smithsonian National Museum of Natural History (USNM), Royal Saskatchewan Museum (RSM) and Royal Alberta Museum (RAB).
(XLS)
